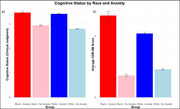# Investigating the Impact of Anxiety and Depression on Cognition Across Racial Groups in Older Adults

**DOI:** 10.1002/alz70860_102225

**Published:** 2025-12-23

**Authors:** Alexandra Doiron, Mahsa Dadar, Cassandra Morrison

**Affiliations:** ^1^ Carleton University, Ottawa, ON, Canada; ^2^ Douglas Mental Health University Institute, Montréal, QC, Canada; ^3^ Department of Psychiatry, McGill University, Montréal, QC, Canada

## Abstract

**Background:**

Older adults who experience anxiety or depression are at a higher risk of progressing to Mild Cognitive Impairment (MCI) or Alzheimer's disease (AD) compared to those who do not. Given that reports of anxiety and depression differ in both incidence and severity among racial groups, it is essential to examine how the relationship between neuropsychiatric experiences and cognitive decline may also differ across races.

**Methods:**

A total of 10,112 participants, including 8182 White and 1390 Black older adults aged 55+ were included from the National Alzheimer's Coordinating Center Data Set to investigate the relationship between anxiety and cognition as well as depression and cognition. The Neuropsychiatric Inventory Questionnaire (NPI‐Q) and the Geriatric Depression Scale (GDS) measured anxiety and depression, respectively. Global cognition and cognitive functioning were quantified using scores from the Clinical Dementia Rating – Sum of Boxes (CDR‐SB) as well as clinical judgement following neuropsychological evaluation. Linear regressions examined the interaction of race with anxiety and depression on cognition. Analyses also controlled for age, sex, years of study, and diagnosis.

**Results:**

Main effects of anxiety and depression were associated with decreased global cognition scores (*p* < .001). The effect of anxiety on global cognition scores increased as a function of severity in both White and Black older adults (*p* < .001). Clinical judgments of cognitive functioning were significantly worse for people with anxiety across both races, but Blacks with anxiety exhibited worse scores across both measures of cognition compared to Whites (*p* < .001; Figure 1). The effect of depression on global cognition did not differ by race. However, higher rates of depression were associated with significantly worse clinical judgments of cognitive functioning in White compared to Black older adults (*p* < .001).

**Conclusion:**

Depression and anxiety are correlated with changes in cognition among both Black and White older adults. The experience of anxiety may have a greater impact on cognitive decline in Black than White adults. White adults with depression appear to exhibit worse cognitive status (as deemed by a clinician) compared to Black adults. These findings suggest differences in how depression and anxiety impact cognitive aging in different racial populations.